# Preparation and Characterization of Emulsion Gels from Whole Faba Bean Flour

**DOI:** 10.3390/foods9060755

**Published:** 2020-06-07

**Authors:** Zhong-Qing Jiang, Jing Wang, Frederick Stoddard, Hannu Salovaara, Tuula Sontag-Strohm

**Affiliations:** 1 Department of Food and Environmental Sciences, University of Helsinki, 00100 Helsinki, Finland; jenni.wang@effem.com (J.W.); hannu.salovaara@helsinki.fi (H.S.); tuula.sontag-strohm@helsinki.fi (T.S.-S.); 2 Department of Agricultural Sciences, Viikki Plant Science Centre and Helsinki Sustainability Centre, University of Helsinki, 00100 Helsinki, Finland; frederick.stoddard@helsinki.fi

**Keywords:** faba bean, protein emulsion gel, starch hydrolysate, gel properties, microstructure, protein resolubilisation, tofu analogue, yogurt analogue

## Abstract

Faba bean protein has good functionalities, but it is little used in the food industry. This study identified a challenge from unfavourable starch gelation when utilizing faba bean for producing protein-based emulsion gel foods, and developed processing methods to overcome that. Two types of protein-based emulsion gel foods, namely yogurt and tofu analogue products, were prepared. The processing methods in this study involved steps of thermal pre-treatment of the beans, dehulling, milling, adding plant oil, homogenization, prevention of starch gelation, and inducing protein gelation. Two methods for preventing starch gelation were studied, namely starch removal and hydrolysis. The gel texture, water-holding capacity, and structural properties of the gel products were evaluated. Both starch-gelation prevention methods produced yogurt and tofu analogue products having typical emulsion gel properties. Hydrolysis of starch was favourable for producing the yogurt analogue, because the hydrolysate compounds improved the gel strength and viscosity. Moreover, it utilized the whole flour, meaning all the nutrients from the cotyledon were used and no side-stream was created. In contrast, starch removal was slightly better than hydrolysis for producing the tofu analogue, because the hydrolysate lowered the gel strength and water-holding capacity of the products. It is both possible and ecologically sustainable to utilize whole faba bean flour for making emulsion gel products.

## 1. Introduction

Faba bean (*Vicia faba L.*) is an important plant protein source, being rich in protein (29% on a dry weight basis [1]) and is grown in cool climates around the world. There is a rapidly growing demand for developing more and better plant protein foods in order to reduce the growth in demand for animal protein foods. For this purpose, pulses are an attractive option owing to their high protein content and low need for nitrogen fertilization, and faba bean is a prime candidate. The major fraction of faba bean protein, globulin, has similarities to soy globulin in molecular structure, thermal stability, solubility, emulsification, and gelling [2,3,4,5], suggesting that faba bean protein should be as versatile and functional for food use as soy protein. Protein stabilized oil-in-water emulsion gel foods made from soy, including tofu and yogurt-like products, are increasingly popular in the market because of their good nutritional value, palatability, texture, and perceived environmental friendliness. Use of legume proteins other than soy is a relatively new area for investigation [4,5] and makes these classes of food available to those with soy allergy and regions where soy does not grow well. There are similarities and differences between tofu and yogurt-like products. For example, soy-based tofu (coagulated by GDL, glucono-delta-lactone) and yogurt-like products are both processed with treatment steps involving protein denaturation and acid-induced coagulation. The coagulation temperature in tofu making is preferably above 60 °C [6], while that in yogurt making is normally below 45 °C [7]. The higher coagulation temperature can make the gel firmer [8,9]. The acidification rate and gelation rate differ in GDL tofu and yogurt, leading to differences in the gel structure and texture [10]. Therefore, studying faba bean yogurt and tofu analogues in parallel can yield extra insights.

Technical difficulties have limited the development of faba bean emulsion gel foods. First, dry faba bean flour contains less than 2% lipid, and aqueous processing reduces this concentration further below the 3% lipid concentration in standard tofu and yogurt products. Lipids play important roles in emulsion gel formation and structure, for example, oil droplets function as a filling material in the emulsion gel. An increase in oil content normally increases the emulsion gel strength [9]. Second, faba beans contain about 40% starch [1], which can gelatinize during the protein denaturation step in emulsion gel production. The gelatinized starch increases the viscosity of the suspension, so it interrupts the further process. Moreover, the gelatinized starch forms gels that are glue-like, paste-like or too stiff, and different from the texture of tofu or yogurt. Third, faba bean contains lipoxygenase and other endogenous enzymes that induce “beany flavour” during wet processing such as emulsion production. This has been recently solved by an optimized heating procedure [11].

Starch gelation can be prevented by physical removal of the starch granules, but this removes some other nutrients as well. Since the starch represents a considerable proportion of the seed, it would be advantageous to keep it in the emulsion gel, forming a firm structure, minimizing nutrient loss, and maximizing the sustainability of the process. For example, gelatinized starch can be hydrolysed with α-amylase to reduce its potential interference in the formation of the protein gel. Starch α-amylase hydrolysis products are a complex group of carbohydrates consisting of oligosaccharides, maltotriose, maltose, glucose, and dextrins [12,13]. These may interact with protein and influence its solubility, emulsifying, and gelling properties. The formation of protein–maltodextrin interaction in whey protein and soy protein through Maillard reaction changed the functional properties of the proteins [14,15,16]. Sugars such as sucrose, glucose, ribose, and lactose affect the gelling properties of proteins including soy protein and caseinate [17,18,19]. Hence, there is a need to study the effects of carbohydrates, especially starch hydrolysis products, on the functional properties of proteins from pulses.

For these reasons, we set out to develop and compare two different processes for producing emulsion gels based on faba bean protein. The two processes differed mainly in the methods for preventing the starch gelation, while they were similar in the other steps including seed pre-treatment, milling, emulsion preparation, cooking, and gelation. The first method for preventing the starch gelation was to remove the starch granules from the flour water suspension by centrifugation. The other method, aimed at utilizing the whole bean without a step for starch separation, was a liquefaction process involving enzymatic hydrolysis of gelatinized starch, resulting in a product with a potentially novel structure having abundant starch hydrolysate compounds incorporated in the protein-based emulsion gel. Therefore, this study aimed to investigate how the starch hydrolysate compounds affect the gel properties in two contrasting types of protein gel systems, namely, yogurt analogue and tofu analogue.

## 2. Materials and Methods

**Figure 1:**
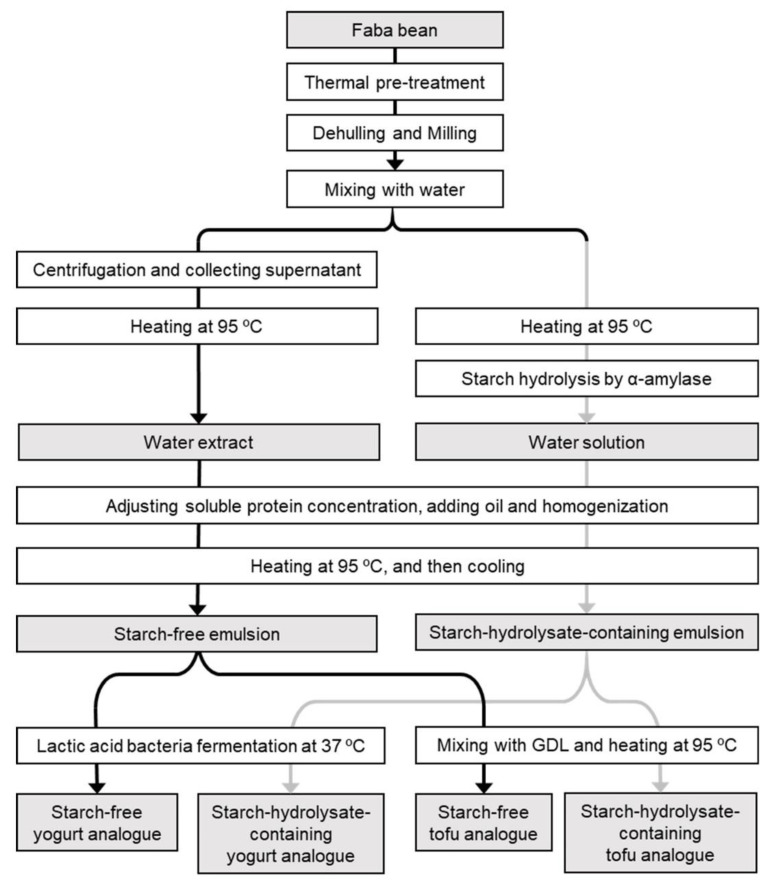
Flow chart of the production methods for faba bean starch-free yogurt analogue, starch-hydrolysate-containing yogurt analogue, starch-free tofu analogue and starch-hydrolysate-containing tofu analogue. GDL, glucono-delta-lactone.

In order to create faba bean emulsion gel structures for food applications, several steps were taken, including (a) heat pre-treatment to denature the flavour-affecting enzymes, (b) dehulling and milling, (c) addition of water and plant oil, (d) homogenization, and (e) protein gelation in combination with prevention of starch gelation (Figure 1).

Faba bean (cultivar ‘Kontu’) was grown and harvested in a Finnish experimental field in 2011 [20]. The flour was prepared as described by Jiang et al. (2016) [11]. Briefly, beans were heated in a microwave for 1.5 min at 950 W, dehulled, and milled using a high-speed mill (Ultra Centrifugal Mill ZM 200, Retsch, Haan, Germany) equipped with a ring sieve having a pore size of 0.5 mm and a rotation speed of 12,000 rpm. Dehulling of the beans was conducted before the aqueous process, because tannins in the hulls cross-link with proteins [1], discolour the solution, and affect the flavour.

### 2.1. Preparation of Emulsion

The flours were mixed with Milli-Q water in a ratio of 1:7 and stirred for 1 h. The suspension was centrifuged at 16,000× *g* (relative centrifugal force) at 5 °C for 20 min. The supernatant was collected, heated at 95 °C for 10 min and labelled as a water extract (heated water extract). The aim of the heating step was to facilitate the protein denaturation, which is crucial for the protein gelation.

Other samples of the flour water suspension were heated at 95 °C using a water bath for 10 min with constant manual stirring. When the cooked suspension cooled to 70 °C, 1 mL of a commercial food-grade thermal stable α-amylase solution (Termamyl^®^Ultra 300 L, Novozyme, Copenhagen, Denmark) was added for each 150 g of flour and the mixture was incubated at 70 °C for 1 h. The mixture was labelled as a water solution (heated water solution). The aim of the heating step in this case was to facilitate the protein denaturation as well as the preparation of starch hydrolysis (the α-amylase reacts with gelatinized starch but not native starch granules). The water extract and water solution were prepared in three independent replicates.

Small portions of the water extract and water solution were centrifuged at 20,238× *g* for 10 min. The soluble protein content in each supernatant was analysed by Lowry’s method [21] using Bio-Rad DC protein assay kits (Bio-Rad Laboratories Inc., Hercules, CA, USA) with bovine serum albumin used as a standard. Then, more water was added to the water extract and the water solution to adjust and equalize their soluble protein content. The desired protein contents were 1.8% and 2.5% for preparing yogurt analogue and tofu analogue, respectively.

Rapeseed oil (Rainbow, Helsinki, Finland) was added to the water extract and water solution to reach a final oil concentration of 3% (*w*/*w*). The mixtures were pre-homogenized using a disperser (IKA^®^ Digital Ultra-Turrax^®^ T-25, Staufen, Germany) at 22,000 rpm for 2 min, followed by continuous and circular homogenization using a valve-type homogenizer (Rannie, APV, Copenhagen, Denmark) at 150–200 bar for 15 min at room temperature. The homogenized liquids were heated at 95 °C in a water bath for 10 min. The cooked oil-in-water emulsions prepared from the water extract and water solution were labelled as “starch-free emulsion” and “starch-hydrolysate-containing emulsion”, respectively.

### 2.2. Yogurt Analogue Gel Preparation by Lactic Acid Bacteria Fermentation

After the two emulsions cooled to room temperature, samples were inoculated with 0.3% lactic acid bacteria (frozen culture, ABT-1, Chr. Hansen, Hørsholm, Denmark) and incubated in closed bottles at 37 °C for 6 h. After overnight storage at 5 °C, the fermented emulsions were stirred and pumped using a plastic syringe in order to produce analogues of stirred yogurt. The gels were labelled as “starch-free yogurt analogue” and “starch-hydrolysate-containing yogurt analogue”, and were stored at 5 °C before further analysis. The yogurt analogues were prepared in three independent replicates.

### 2.3. Tofu Analogue Gel Preparation by Glucono-Delta-Lactone (GDL) Coagulation

The cooked emulsions were degassed by a motor-driven vacuum pump, cooled to room temperature, poured into glass containers, and then mixed with GDL solution. A range of GDL concentrations from 0% to 1% (*w*/*v*) was tested for its effect on pH and protein solubility and a concentration of 0.4% was selected for preparing the tofu analogue. After the GDL was well mixed with the emulsions, they were heated in an oven (Termaks, Bergen, Norway) at 95 °C for 40 min and stored overnight at 5 °C to produce “starch-free tofu analogue” and “starch-hydrolysate-containing tofu analogue”. The tofu analogues were prepared in three independent replicates.

### 2.4. Sensorial Observation

The appearance, smell, and taste of the emulsions and gels were observed by at least three researchers, who have adequate knowledge in the field.

### 2.5. Soluble Protein Content and pH in Suspensions Containing GDL

Portions of the water extract and the water solution with soluble protein concentration of 2.5%, without added oil, were homogenized in the same way as for the emulsions, and then heated at 95 °C in a water bath for 10 min. After the solutions cooled to room temperature, they were mixed with GDL to reach a GDL concentration between 0 and 1% (*w*/*v*), and heated at 95 °C in an oven for 40 min. After overnight storage at 5 °C, the mixtures were stirred, and their pH was measured. The mixtures were centrifuged at 20,238× *g* for 10 min. The soluble protein content in the supernatant was analysed with Lowry’s method as described above.

### 2.6. Microstructure Observation with Light Microscopy

The water extract, the water solution, and two emulsions were observed at 40× and 100× magnifications using an optical microscope (Axio Lab. A1, Carl Zeiss, Oberkochen, Germany) equipped with a digital camera.

The microstructure of the tofu analogue gels was observed with the same optical microscope after a sequential fixing and staining, with a method adopted from [22] and [23] with modifications. A cube of gel, 1 cm on each side, was frozen in liquid nitrogen. A slice (about 30 μm thickness) of the frozen sample was cut using a microtome cryostat (Leica 2800 Frigocut-E Microtome Cryostat, Reichert-Jung, New York, NY, USA) at −20 °C, collected on a cover glass and melted. Then several drops of fat stain containing Oil red O was added to the sample and allowed to stain for 30 s. Next, a few drops of protein stain containing Coomassie Brilliant Blue was added to the sample and allowed to stain for 1 min. Finally, the stain was gently rinsed with Milli-Q water and the sample was observed in the microscope.

### 2.7. Total Solid Content in the Gels

The solid contents in all the analogue gels were analysed according to [24] with modifications. Ten grams of the gel samples were dried in beakers in an oven at 95 °C for 8 h. The weight of remaining solid was measured for calculating the total solid content. Three replicates were measured.

### 2.8. Water-Holding Capacity (WHC) of the Gels

The water-holding capacities of all the analogue gels were analysed as described by [25] with slight modifications. Each gel for analysis was coagulated directly in a centrifuge tube (10 g yogurt analogue gel in a 15 mL centrifuge tube, and 30 g tofu analogue gel in a 50 mL centrifuge tube), and then centrifuged at 2057× *g* at room temperature for 30 min. The aqueous solution that was not held by the gel and moved to the top of the gel was carefully collected and air-dried at 95 °C for 8 h. The amount of the evaporated water was regarded as actual released water (W_r_). The total amount of water in sample (W_t_) was calculated from the total solid content. The water-holding capacity (WHC) was calculated as the percentage of the water held in the gel structure (W_t_-W_r_) in the total amount of water (W_t_). WHC was measured in three replicates.

### 2.9. Viscosity Measurement

The viscosity of the water extract and the water solution (1.8% soluble protein content), the emulsions made from them and the yogurt analogues were measured using a ThermoHaake RheoStress 600 rheometer (Thermo Electron GmbH, Dreieich, Langenselbold, Germany) equipped with cone-and-plate geometry (35 mm diameter, 2° angle, 0.104 mm gap, and 10 °C measurement temperature), in three replicates. In order to measure the shear-rate-shear-stress curve of the samples, the shear rate was increased from 0.3 s^−1^ to 100 s^−1^, kept at 100 s^−1^ for 10 s, and finally decreased to 0.3 s^−1^.

### 2.10. Gel Property Analyses

Frequency-sweep analyses were conducted on the gels using the ThermoHaake RheoStress 600 rheometer. The yogurt analogues were analysed using cone-and-plate geometry (35 mm diameter, 2° angle, 0.104 mm gap, and 10 °C measurement temperature). The yogurt analogue was loaded onto the plate using a plastic soft straw dropper. The stress-sweep oscillation test was conducted with an increasing stress between 0.5 Pa and 100 Pa and a constant frequency of 1 Hz on the sample to determine the linear viscoelastic region. The gel sample was then reloaded and analysed with frequency-sweep oscillation test with an increasing angular frequency from 0.6 rad/s to 100 rad/s and a constant stress of 1 Pa. The frequency-sweep tests were conducted on three replicates of each yogurt analogue.

### 2.11. Textural Analysis

The textural properties of the tofu analogues were analysed using a texture analyser (TA; TA-XT 2i, Stable Micro Systems Ltd., Godalming, England) equipped with a 36 mm cylindrical probe (SMSP/36R) at room temperature. The analysed gel samples with a weight of 100 g were prepared in cylindrical glass cups with an inner diameter of 79 mm. A single compression test was conducted, and the probe penetrated the sample at a test speed of 2 mm/s until 40% of strain was reached. Six gels of each sample were prepared and measured independently.

### 2.12. Protein Resolubilisation from Tofu Analogues

Tofu analogues were mixed with one of the following five solutions: Milli-Q water, 2% sodium dodecyl sulfate (SDS), 10 mM dithiothreitol (DTT), 2% SDS + 10 mM DTT, and 8 M urea, at a ratio of 1:10 (*w*/*w*), at 25 °C for 20 h. Similarly, the cooked emulsions were mixed with 2% SDS + 10 mM DTT. The mixtures were centrifuged at 20,238× *g* at room temperature for 15 min. The protein content in the supernatant was analysed using Bio-Rad DC protein assay kits as previously described. The protein resolubility was calculated as the percentage of the protein solubilised from the gel by each solution in the protein solubilised from the emulsion by the 2% SDS + 10 mM DTT solution. The measurements were conducted in three independent replicates.

### 2.13. Statistical Analysis

The replications of the sample preparations and analyses were described above in each section. Means and standard deviations of all replicates were calculated. OriginPro 8.6 software (OriginLab Corporation, Northampton, MA, USA) was used to conduct one-way analysis of variance (ANOVA) with Tukey’s test at a 95% confidence level.

## 3. Results and Discussion

With the processes described above, yogurt analogue and tofu analogue were successfully produced from both whole faba bean flour and a starch-free fraction. The flavour of the tofu analogues was bland, while the yogurt analogues were slightly acidic, and both were considered to be palatable by the evaluation panels. The colour and opacity of the faba bean tofu analogues and yogurt analogues were similar to those of soy-based tofu and commercial soy-based yogurt analogue. Furthermore, the intermediate products made during the process, the emulsions, were similar in appearance to soy-based milk analogues.

The solid contents of the starch-free and hydrolysate-containing yogurt analogues were 5.6 ± 0.1% and 11.4 ± 1.3% (mean ± standard deviation), respectively, and those of the starch-free and the hydrolysate-containing tofu analogues were 9.0 ± 0.1% and 16.3 ± 0.6%, respectively. The differences in solid contents in the presence of similar protein and fat contents were attributable to the loss of water-insoluble components during starch removal, and the increase in the solid content due to the starch hydrolysate. The tofu analogues had higher solid contents than the yogurt analogues because they had a lower dilution ratio at the step of protein content adjustment during the process.

### 3.1. Rheological Properties of Yogurt Analogues

**Figure 2:**
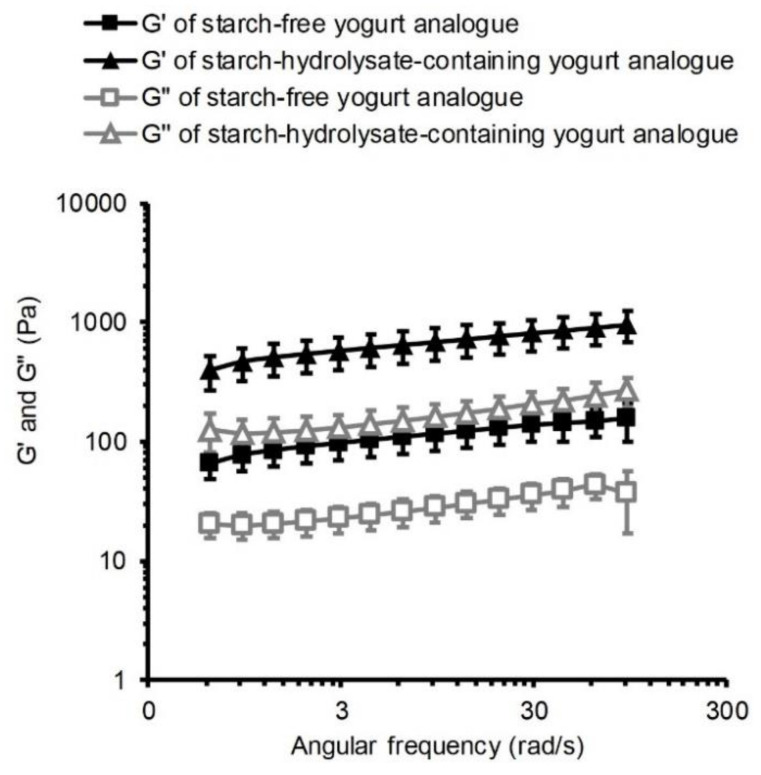
Viscoelasticity (storage modulus, G’, and loss modulus, G’’) of faba bean starch-free and starch-hydrolysate-containing yogurt analogues measured by frequency-sweep oscillation. Each point represents the average value of triplicate measurements with standard deviations.

**Figure 3:**
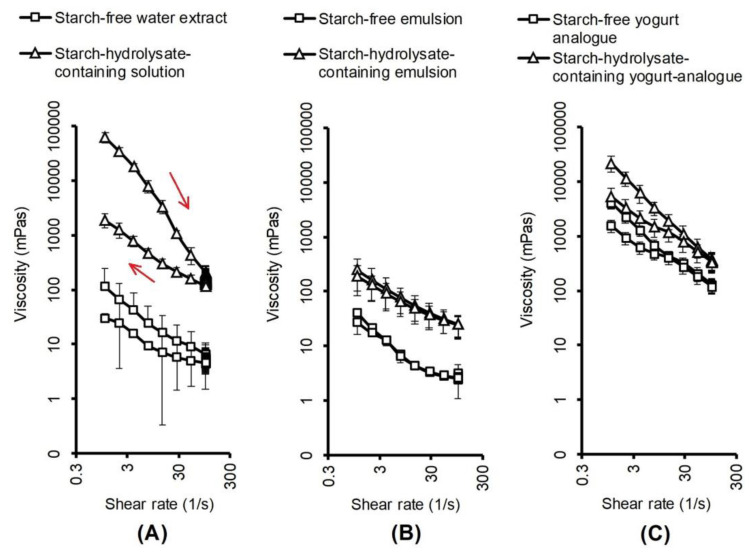
Viscosity of faba bean starch-free water extract (1.8% soluble protein content), and starch-hydrolysate-containing solution (1.8% soluble protein content) (**A**), the emulsions made from these solutions (**B**) and the yogurt analogues (**C**). Each point represents the average value of triplicate measurements with standard deviations. The arrows in the figure indicate the order of the data points of the starch-hydrolysate-containing solution. The viscosity in the up measurement (shear rate increasing) was higher than that in the down measurement (shear rate decreasing). The other curves had similar order.

The yogurt analogues produced from the starch-free and the starch-hydrolysate-containing emulsions both had typical solid gel properties, as shown by the viscoelasticity analysis results (Figure 2), where the storage modulus, G’, was significantly higher than the loss modulus, G’’. The viscosity measurements (Figure 3C) revealed some typical characteristics of weak gels such as shear thinning and hysteresis properties. Similarly, stirred dairy yogurts had higher storage modulus than loss modulus along with shear thinning and hysteresis properties [26].

The starch-hydrolysate-containing yogurt analogue was stronger than the starch-free one, as it had higher firmness and viscosity revealed by its higher storage modulus and higher loss modulus values (Figure 2). The higher viscosity of the starch-hydrolysate-containing yogurt analogue was also shown by the results of the shear-viscosity analysis (Figure 3C). Many other previous attempts have been made to improve yogurt gel strength by increasing protein content [27], enzymatic treatment [28], and adding food hydrocolloids [29]. Therefore, it is advantageous that the starch-hydrolysate-containing yogurt analogue can form a strong gel without additional protein or added hydrocolloids.

The solutions, emulsions, and gels at different steps of the production of the yogurt analogues all showed shear-thinning properties (Figure 3). The emulsions were less viscous than the solutions, which is attributable to the homogenization process involved in the emulsion production. The viscosity hysteresis gaps of the emulsions were much smaller than those of the solutions. The yogurt analogues were much more viscous than the emulsions, which is attributable to the acidic coagulation of proteins induced by lactic acid bacteria fermentation and the formation of protein–protein interactions.

The viscosities of the starch-hydrolysate-containing solutions, emulsions and gels were all higher than those of the starch-free equivalents (Figure 3). The hydrolysis products of starch released by α-amylase consist of oligosaccharides, maltotriose, maltose, glucose, and dextrins, which are much smaller and less viscous than starch molecules [12,13], but still have sites that can form hydrogen bonds. In particular, the molecular weight of dextrin is still high, and it can cause high viscosity and even form thermally reversible gels [12]. Similarly, glucose, lactose and sucrose addition all increased the viscosity of soy protein isolate dispersions, with more effectiveness when the dispersions were heat-denatured [18].

The starch-hydrolysate-containing water solution had a clear hysteresis of viscosity recovery after shear thinning, and its hysteresis gap was larger than that of the starch-free water extract (Figure 3A). In addition, the decrease of viscosity after homogenization was much larger in the starch-hydrolysate-containing water solution than in the starch-free water extract (Figure 3A,B). These differences are mainly attributable to the presence of the starch hydrolysate compounds that form hydrogen bonds, create steric interactions and, hence, induce high viscosity in solutions before being sheared or homogenised.

The viscosity of the starch-free yogurt analogue gel was 100-fold higher than that of the emulsion, and that difference was larger than that in the starch-hydrolysate-containing system (80-fold increase) (Figure 3B,C). This difference may be attributable to the starch hydrolysate compounds interfering with and restricting the formation of protein–protein interactions. Previous studies have also found that sucrose addition could retard protein gelation by increasing the viscosity and decreasing the protein collision frequency [18,30].

### 3.2. Firmness of Tofu Analogues

**Figure 4:**
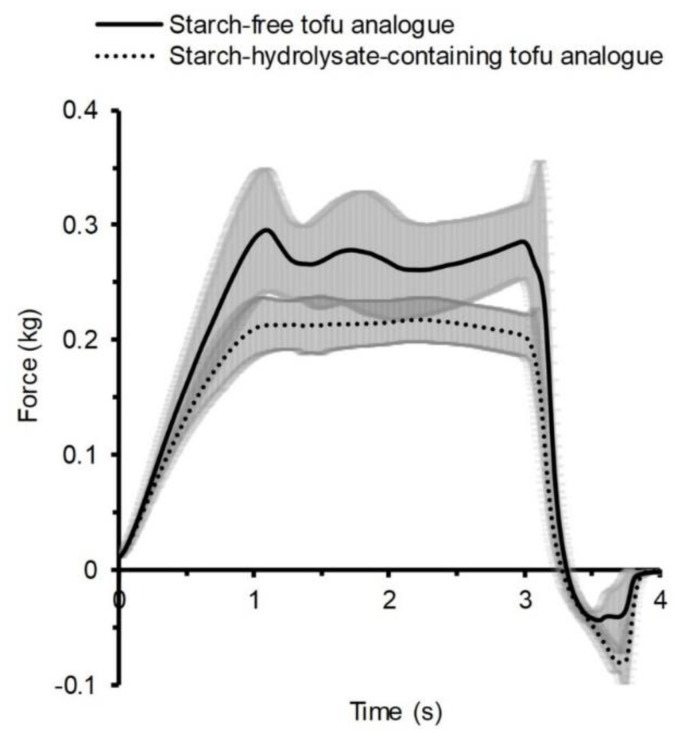
Textural properties of faba bean starch-free and starch-hydrolysate-containing tofu analogues measured by a compression test. The shaded areas show the standard deviation from six replicates.

As shown in the textural analysis results, the firmness properties of the starch-free and starch-hydrolysate-containing tofu analogues were similar in general (Figure 4). The starch hydrolysate compounds did not alter the gel strength in the tofu analogues as they did in the yogurt analogues. The starch-free tofu analogue had a clearly higher firmness than the starch-hydrolysate-containing tofu analogue, as it had a higher resistance force during the test. The time force curve of the starch-free tofu analogue had a steep increase before and a slight decrease after reaching its first peak point. This peak represented a “surface breakage” event during the measurement, demonstrating that the sample surface was intact and strong. The time force curve of the starch-hydrolysate-containing tofu analogue lacked the “surface breakage” event, showing that its structure had poorer integrity. The starch-hydrolysate-containing tofu analogue had a larger pulling force against the analysis probe withdrawing movement than the starch-free tofu analogue did, which indicated a greater stickiness. Normally, tofu-type products are expected to have a sufficient firmness that can maintain their integrity during different movements and provide a “chewy” mouth-feel. An intact and firm surface is also favourable for tofu-type products, while high stickiness is unfavourable. Similar studies have shown that the addition of either glucose, lactose, or sucrose decreased the storage modulus and gel hardness of GDL-induced soy protein gel [18], and sucrose addition decreased the gel rigidity (complex modulus) of a NaCl-induced gel of heat denatured whey protein [30].

### 3.3. Water-Holding Capacity (WHC) of the Gels

The water-holding capacity (WHC) of the starch-free tofu analogue (63 ± 3%, mean ± standard deviation) was slightly higher than that of the starch-hydrolysate-containing tofu analogue (55 ± 2%; *p* < 0.05). On the other hand, the WHC values of the yogurt analogues did not differ significantly (61 ± 6% for starch-free yogurt analogue and 50 ± 5% for starch-hydrolysate-containing yogurt analogue, *p* > 0.05, *n* = 3). The presence of the starch-hydrolysate compounds was associated with decreased WHC of the gel, although they are usually considered hydrophilic.

A low WHC is often regarded as an indicator of a weak gel structure that cannot effectively trap the water molecules [31,32]. This agrees with the previous results in this study that the presence of starch-hydrolysate compounds weakened the gel firmness of the tofu analogue. The starch hydrolysate compounds could further reduce the protein–water interaction by competing with the proteins for water, reducing the overall water-holding power of the gel dominated by the protein–protein crosslinking network. In contrast, the addition of glucose, lactose, and sucrose significantly increased the WHC of GDL-induced soy protein gels [18], although those sugars decreased the strength of the gels.

### 3.4. Microstructure of the Emulsions

**Figure 5:**
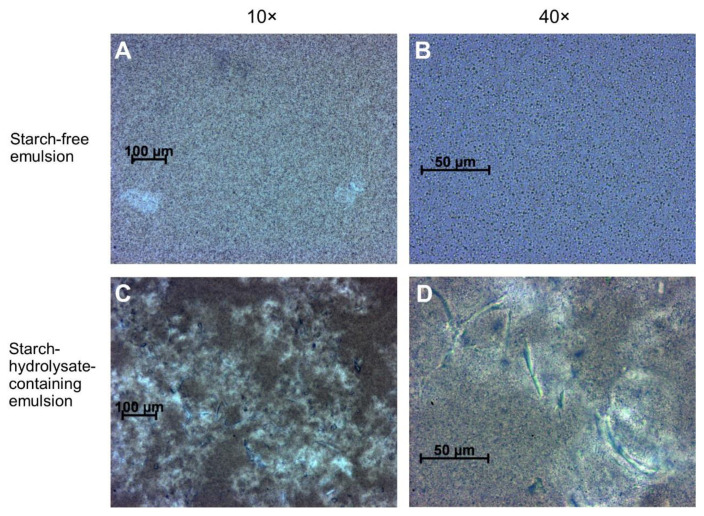
Microstructure of faba bean starch-free emulsions (**A**,**B**) and starch-hydrolysate-containing emulsions (**C**,**D**) observed in an optical microscope with 10× (**A**,**C**) and 40× (**B**,**D**) objectives.

The starch-free and starch-hydrolysate-containing emulsions both had typical oil-in-water emulsion type microstructure (Figure 5). The oil droplets were globular with diameters around 1 µm. As shown in Figure 5C, the starch-hydrolysate-containing emulsions had some rod-shaped particles (length up to 100 µm) consistent with fragments of plant cell wall. These fragments did not appear in the starch-free emulsions, because they were removed by centrifugation during the emulsion preparation. The cell wall fragments in the starch-hydrolysate-containing emulsion were not broken down by the homogenization process during the emulsion preparation step, although the homogenization process was effective enough to micronize the oil particles. As a result, these cell wall fragments may sterically interfere with the continuity of the networking structure of starch-hydrolysate-containing tofu analogue gels, and may be responsible for the decreases in their gel firmness (Figure 4) and water holding capacity (WHC).

### 3.5. Microstructure of the Tofu Analogues

**Figure 6:**
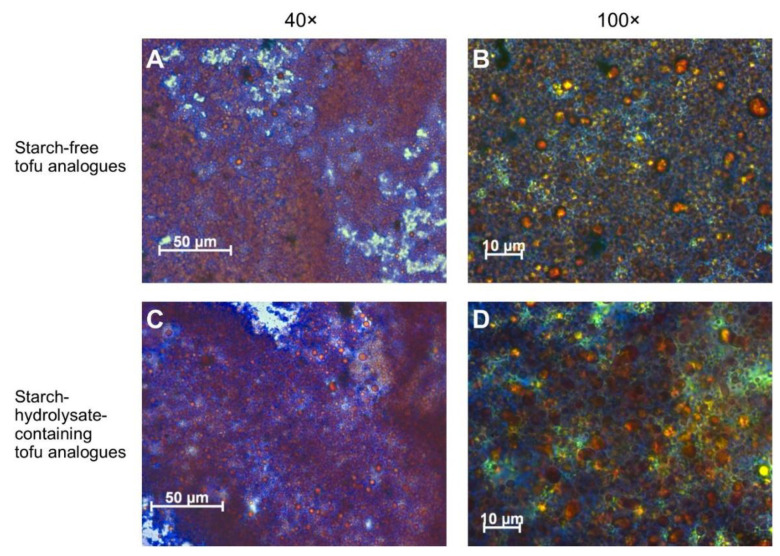
Microstructure of faba bean starch-free tofu analogues (**A**,**B**) and starch-hydrolysate-containing tofu analogues (**C**,**D**) observed in an optical microscope with 40× (**A**,**C**) and 100× (**B**,**D**) objectives. The lipids were stained red by Oil red O and the proteins were stained blue using Coomassie Brilliant Blue.

The microstructure of the starch-free and the starch-hydrolysate-containing tofu analogues had only minor differences (Figure 6). They both had a typical emulsion gel structure, in which the proteins (stained blue) formed a continuous network. The globular oil droplets (stained orange) were covered by the protein layers. The protein coverage on the surface of the oil droplets in the starch-free tofu analogue was slightly more continuous and intact than that in the starch-hydrolysate-containing tofu analogue.

### 3.6. Protein Trapping forces in Tofu Analogues

**Figure 7:**
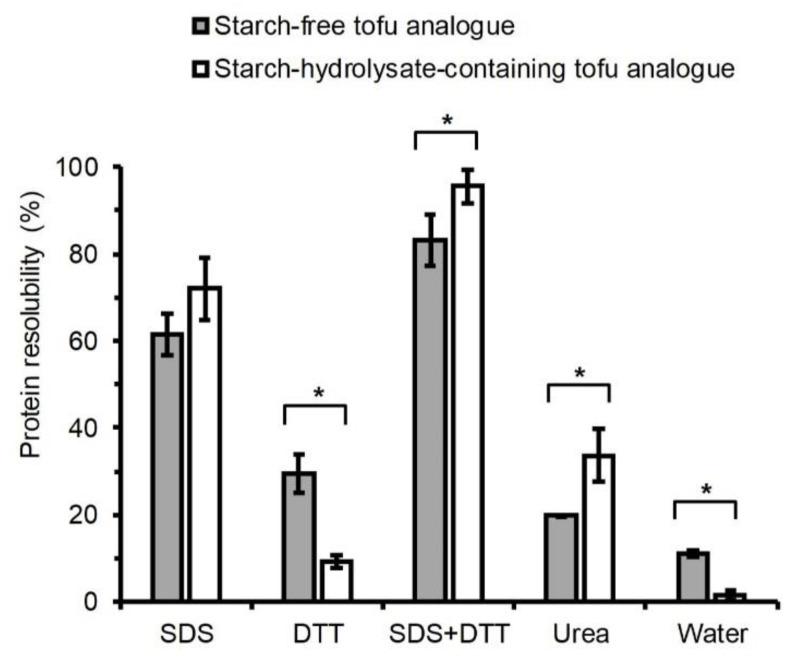
Resolubility of proteins from starch-free and starch-hydrolysate-containing tofu analogues in Milli-Q water and in 2% sodium dodecyl sulfate (SDS) solution, 10 mM Dithiothreitol (DTT) solution, 2% SDS + 10 mM DTT solution and 8 M urea solution. Each result shows the average of three measurements with the standard deviation. * indicates significant difference between two types of tofu analogues (*p* < 0.05).

There are different types of molecular interaction forces that can protect the tofu analogues from being dissolved and deformed in water. Those forces mainly protect the proteins that form the gel network from being solubilised in water, so they were regarded as the protein-trapping forces in this study. The profiles of protein resolubilisation in different reagents were clearly different between the starch-free and starch-hydrolysate-containing tofu analogues, which indicated differences in the profile of protein trapping forces (Figure 7). A 2% SDS solution can break the non-covalent interactions including hydrogen bonds, electrostatic interaction, hydrophobic interaction, and Van der Walls force; 10 mM DTT solution can break intermolecular disulfide bonds; the combination can break non-covalent interactions along with inter- and intramolecular disulfide bonds; and 8 M urea solution can break hydrophobic interactions and hydrogen bonds [33,34,35,36,37].

During the water solubilisation test, only 11% and 2% protein were extracted from the starch-free and the starch-hydrolysate-containing tofu analogue samples, respectively (Figure 7). Tofu gels having lower water-solubility of proteins are more desirable, because they do not dissolve or disintegrate during cooking in water [18]. These results also demonstrated that the starch-hydrolysate-containing tofu analogue had more protein-trapping forces than the starch-free tofu analogue.

Solutions containing 2% SDS + 10 mM DTT had the most effective solubilising power, extracting about 90% of the protein from the tofu analogues (Figure 7). This indicated that the combination of non-covalent interactions, intermolecular disulfide bonds, and intramolecular disulfide bonds contributed to the most protein trapping forces in those samples.

The solution containing 2% SDS alone extracted about 70% of the proteins from both tofu analogues (Figure 7), while the solution containing 10 mM DTT alone extracted less than 30% of the proteins. These results showed that the protein trapping forces of the samples were mostly non-covalent interactions, rather than intermolecular disulfide bonds. And the proportions of non-covalent interactions among the total protein trapping forces were similar in the starch-free and starch-hydrolysate-containing tofu analogues.

The 10 mM DTT solution extracted more proteins from the starch-free tofu analogue than from the starch-hydrolysate-containing tofu analogue. After subtracting the water-extractable proteins, the amount of proteins specifically trapped by intermolecular disulfide bonds were around 19% and 8% in the starch-free and starch-hydrolysate-containing tofu analogues, respectively.

The 8 M urea solution extracted less protein from the starch-free tofu analogue than from the starch-hydrolysate-containing tofu analogue (Figure 7). After subtracting the water-extractable proteins, the amount of proteins specifically trapped by hydrophobic interaction and hydrogen bonds were 9% and 28% in the starch-free and starch-hydrolysate-containing tofu analogues, respectively, indicating that the proportion of hydrophobic interaction among the total protein trapping forces was correspondingly lower. Similarly, a buffer containing SDS, urea and 2-mercaptoethanol solubilised more than 90% of soy protein from a GDL-induced gel, whereas an SDS-urea buffer solubilised 54–68% protein, and the addition of sugar increased the solubility of soy protein in SDS-urea buffer as well as in water [18].

The serial protein-solubilisation test showed that the starch-hydrolysate-containing tofu analogue contained more protein-trapping forces (hydrophobic interactions and hydrogen bonds) than the starch-free tofu analogue. This finding was in agreement with the larger hysteresis gap in the starch-hydrolysate-containing solution than the starch-free solution in the viscosity analysis (Figure 3). This suggests that the hydrogen bonds between proteins may be enhanced by the presence of the starch hydrolysate, or that the proteins are held by hydrogen bonds between the proteins and the starch hydrolysate compounds.

### 3.7. Relationship between GDL (Glucono-Delta-Lactone) Concentration, pH and Protein Solubility

**Figure 8:**
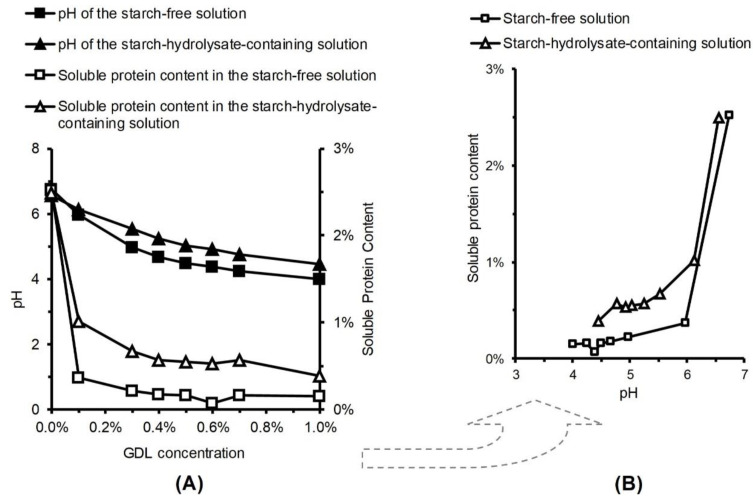
The pH and soluble protein content of the faba bean starch-free and starch-hydrolysate-containing solutions having different concentration of glucono-delta-lactone (GDL) (**A**). The same data was replotted to show the relationship between the soluble protein content and pH (**B**). Each point shows the average of duplicate measurements.

The starch-free and starch-hydrolysate-containing water solutions originally had similar pH values, around 6.6 (Figure 8A). As the GDL concentration increased from zero to one percent, the pH of the starch-free solution decreased to about 4.0, while the pH of the starch-hydrolysate-containing solution decreased to about 4.4. The difference may be due to the buffering capacity provided by the greater solids content and hydrophilic sites of the starch hydrolysate.

As the pH decreased, the protein solubility decreased in both the starch-free and the starch-hydrolysate-containing solutions (Figure 8B) because of protein isoelectric precipitation. The solubility of faba bean protein has been reported to be lowest at pH 4.5 [38]. The soluble protein content decreased more steeply in the starch-free solution than in the starch-hydrolysate-containing solution (Figure 8B). This difference could be partly attributable to the role of the starch hydrolysate compounds in increasing the viscosity of the solution (Figure 3), thus forming steric barrels between the protein molecules and limiting their mobility and tendency of aggregation and precipitation.

In summary, the starch hydrolysate compounds enhanced the gel strength of the yogurt analogue but reduced that of the tofu analogue. The effect on the yogurt analogue could be associated with the effects on increased hydrogen bonding, solid content, and viscosity. Moreover, starch hydrolysate compounds may form a weak gel that is compatible and significantly beneficial for the yogurt analogue system [12]. On the other hand, their detrimental effects on the gel strength of the tofu analogues can be attributed to their effect on increased viscosity that limited the mobility and collision frequency of the protein molecules, thus retarding the protein gelation, and to their role in steric hindrance interrupting the formation of protein–protein interactions, especially disulfide bonds. Furthermore, the starch hydrolysate compounds could prevent the proteins from completing denaturation, as the addition of sucrose [39], lactose, and ribose [19] increased the temperature requirement for whey protein denaturation.

Thus, the starch-hydrolysis method was superior for producing faba bean yogurt analogues, as it increased yield, improved gel strength and viscosity, utilized all of the flour and its nutrients, and avoided a fractionation step. This method, however, produced inferior tofu analogues due to reductions in gel strength and water-holding capacity, so further development is necessary to improve the gel properties of a starch-hydrolysate-containing tofu analogue.

## 4. Conclusions

The production of a “whole flour” emulsion gel food, as a new way of utilizing faba bean, was proven as technologically viable. A process comprising cooking, α-amylase hydrolysis, oil addition, homogenization, and lactic acid bacteria fermentation was proven effective and advantageous in producing the whole-bean yogurt analogues where the proteins effectively stabilised the emulsion and formed a typical gel network structure. The starch hydrolysate compounds improved the gel strength and viscosity of the yogurt analogues. The production of faba bean tofu analogues was also successful, but the starch removal was required in order to maximize gel texture and water-holding capacity. The adverse effects of the starch hydrolysate compounds were attributed mainly to their high viscosity and steric hindrance of proteins. It is likely that these processes will have similar outcomes with other starch-containing grain legumes and there are realistic prospects for producing yogurt analogues from whole flour of faba bean and other pulses in the near future. The development of whole-flour tofu analogues from starch-containing pulses requires further work, but starch-free tofu analogues are feasible already.
